# 2-Methyl-2-phenyl-1-(pyrrolidin-1-yl)propan-1-one

**DOI:** 10.1107/S1600536813009975

**Published:** 2013-04-20

**Authors:** Dong-mei Ren

**Affiliations:** a Security and Environment Engineering College, Capital University of Economics and Business, Beijing 10070, People’s Republic of China

## Abstract

In the title compound, C_14_H_19_NO, the dihedral angle between the benzene ring and the plane of the amide group is 80.6 (1)°. In the crystal, mol­ecules are connected *via* weak C—H⋯O hydrogen bonds, forming chains along the *c*-axis direction. The conformation of the five-memebred ring is an envelope, with one of the ring C atoms adjacent to the ring N atom as the flap atom.

## Related literature

For background to the applications of the title compound as an intermediate in organic synthesis, an important organic synthesis inter­mediate, see: Richard *et al.* (2001 ▶). For the synthetic procedure, see: Richard *et al.* (1995 ▶). For bond-length data, see: Allen *et al.* (1987 ▶).
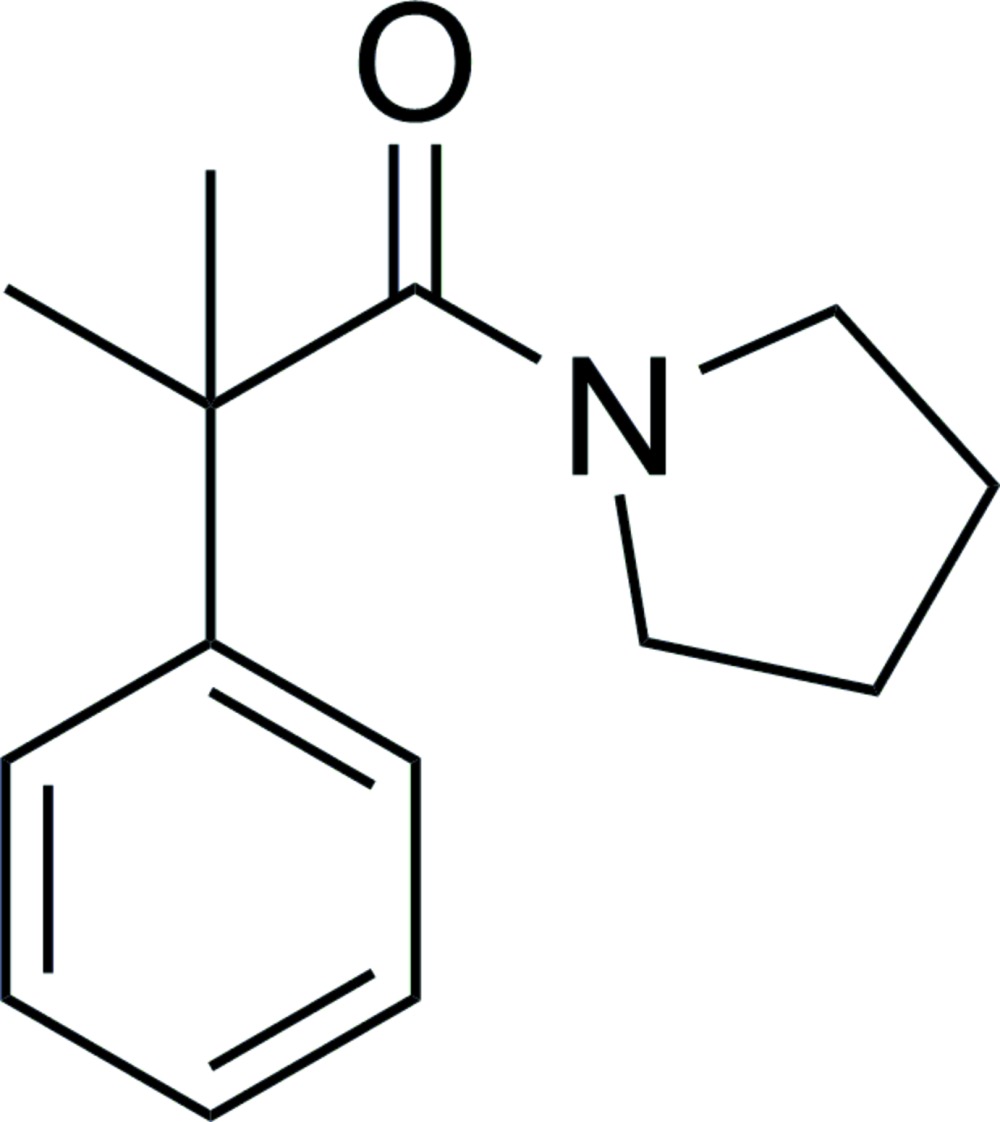


## Experimental

### 

#### Crystal data

C_14_H_19_NO
*M* *_r_* = 217.30
Monoclinic, 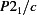

*a* = 8.2330 (16) Å
*b* = 12.534 (3) Å
*c* = 12.192 (2) Å
β = 97.96 (3)°
*V* = 1246.0 (4) Å^3^
*Z* = 4
Mo *K*α radiation
μ = 0.07 mm^−1^
*T* = 293 K
0.30 × 0.20 × 0.10 mm

#### Data collection

Enraf–Nonius CAD-4 diffractometer
Absorption correction: ψ scan (North *et al.*, 1968 ▶) *T*_min_ = 0.979, *T*_max_ = 0.993
2283 measured reflections
2283 independent reflections
1316 reflections with *I* > 2σ(*I*)
*R*_int_ = 0.000
3 standard reflections every 200 reflections intensity decay: 1%

#### Refinement

*R*[*F* ^2^ > 2σ(*F* ^2^)] = 0.063
*wR*(*F* ^2^) = 0.152
*S* = 1.00
2283 reflections
147 parameters
H-atom parameters constrained
Δρ_max_ = 0.17 e Å^−3^
Δρ_min_ = −0.22 e Å^−3^

### 

Data collection: *CAD-4 Software* (Enraf–Nonius, 1985 ▶); cell refinement: *CAD-4 Software*; data reduction: *XCAD4* (Harms & Wocadlo, 1995 ▶); program(s) used to solve structure: *SHELXS97* (Sheldrick, 2008 ▶); program(s) used to refine structure: *SHELXL97* (Sheldrick, 2008 ▶); molecular graphics: *SHELXTL* (Sheldrick, 2008 ▶); software used to prepare material for publication: *SHELXTL*.

## Supplementary Material

Crystal structure: contains datablock(s) I, global. DOI: 10.1107/S1600536813009975/bq2384sup1.cif

Structure factors: contains datablock(s) I. DOI: 10.1107/S1600536813009975/bq2384Isup2.hkl

Supplementary material file. DOI: 10.1107/S1600536813009975/bq2384Isup3.cml

Additional supplementary materials:  crystallographic information; 3D view; checkCIF report

## Figures and Tables

**Table 1:** Hydrogen-bond geometry (Å, °)

*D*—H⋯*A*	*D*—H	H⋯*A*	*D*⋯*A*	*D*—H⋯*A*
C1—H1*A*⋯O1^i^	0.97	2.58	3.510 (4)	160

Symmetry code: (i) 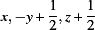.

**Fig. 1.**
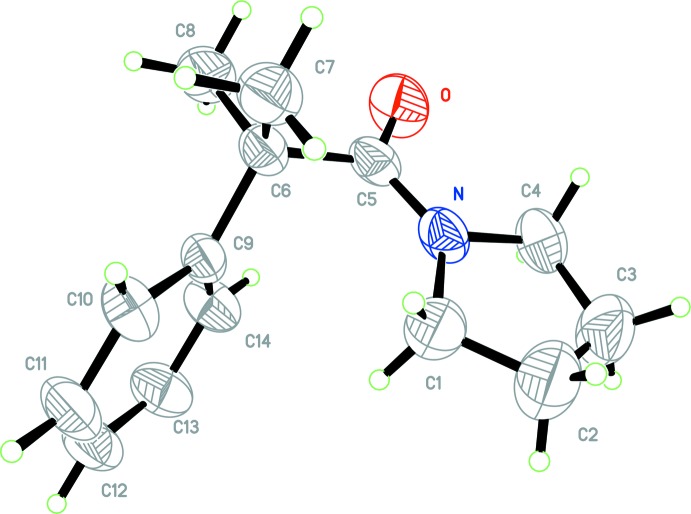
The molecular structure of (I), with the atom-numbering scheme. Displacement ellipsoids are drawn at the 50% probability level.

**Fig. 2.**
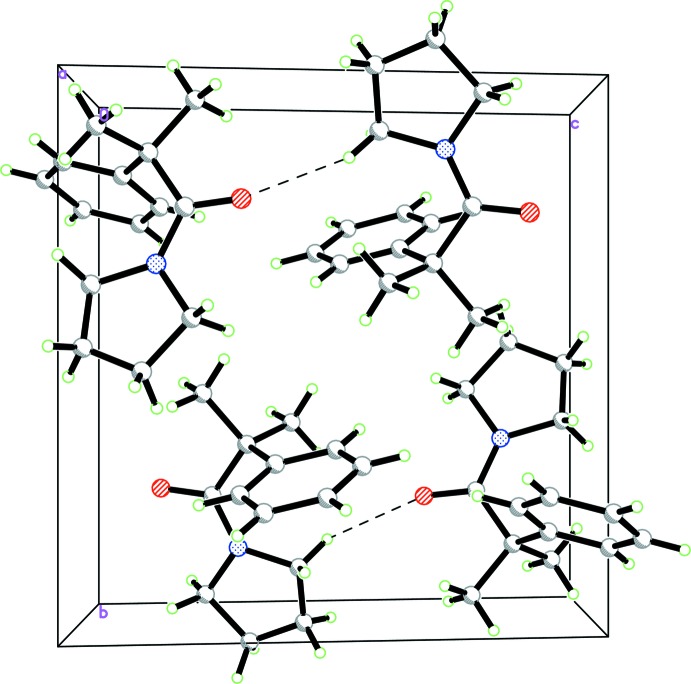
A packing diagram of (I) showing C-H···O bonds with dashed lines.

## supplementary crystallographic information

### Comment

The title compound is an important intermediate in the synthesis of [(piperidinoalkanoyl)phenyl]propionates, which can be utilized to synthesize antihistaminics. And we report here the crystal structure of the title compound, (I).

The molecular structure of (I) is shown in Fig. 1. There is a intermolecular contact C—H···O in the title compound, forming molecular chains along *c* axis direction (Table 1, Fig. 2). The dihedral angles between the benzene ring and the plane of amide is 80.6 (1)°.

### Experimental

The title compound, (I) was prepared by a method reported in literature (Richard *et al.*, 1995). The crystals were obtained by dissolving (I) (0.1 g) in methanol (30 ml) and evaporating the solvent slowly at room temperature for about 8 d.

### Refinement

All H atoms were positioned geometrically and constrained to ride on their parent atoms, with C—H = 0.93, 0.97 and 0.96 Å for aromatic, methylene and methyl H's, respectively. The *U*_iso_(H) = *xU*_eq_(C), where *x* = 1.2 for aromatic H and *x* = 1.5 for other H.

### Crystal data

**Table:**

C_14_H_19_NO	*F*(000) = 472
*M**_r_* = 217.30	*D*_x_ = 1.158 Mg m^−^^3^
Monoclinic, *P*2_1_/*c*	Mo *K*α radiation, λ = 0.71073 Å
Hall symbol: -P 2ybc	Cell parameters from 25 reflections
*a* = 8.2330 (16) Å	θ = 10–13°
*b* = 12.534 (3) Å	µ = 0.07 mm^−^^1^
*c* = 12.192 (2) Å	*T* = 293 K
β = 97.96 (3)°	Block, colorless
*V* = 1246.0 (4) Å^3^	0.30 × 0.20 × 0.10 mm
*Z* = 4	

### Data collection

**Table:**

Enraf–Nonius CAD-4 diffractometer	1316 reflections with *I* > 2σ(*I*)
Radiation source: fine-focus sealed tube	*R*_int_ = 0.000
Graphite monochromator	θ_max_ = 25.4°, θ_min_ = 2.3°
ω/2θ scans	*h* = −9→9
Absorption correction: ψ scan (North *et al.*, 1968)	*k* = 0→15
*T*_min_ = 0.979, *T*_max_ = 0.993	*l* = 0→14
2283 measured reflections	3 standard reflections every 200 reflections
2283 independent reflections	intensity decay: 1%

### Refinement

**Table:**

Refinement on *F*^2^	Primary atom site location: structure-invariant direct methods
Least-squares matrix: full	Secondary atom site location: difference Fourier map
*R*[*F*^2^ > 2σ(*F*^2^)] = 0.063	Hydrogen site location: inferred from neighbouring sites
*wR*(*F*^2^) = 0.152	H-atom parameters constrained
*S* = 1.00	*w* = 1/[σ^2^(*F*_o_^2^) + (0.060*P*)^2^ + 0.270*P*] where *P* = (*F*_o_^2^ + 2*F*_c_^2^)/3
2283 reflections	(Δ/σ)_max_ < 0.001
147 parameters	Δρ_max_ = 0.17 e Å^−^^3^
0 restraints	Δρ_min_ = −0.22 e Å^−^^3^

### Special details

**Table:**

Geometry. All e.s.d.'s (except the e.s.d. in the dihedral angle between two l.s. planes) are estimated using the full covariance matrix. The cell e.s.d.'s are taken into account individually in the estimation of e.s.d.'s in distances, angles and torsion angles; correlations between e.s.d.'s in cell parameters are only used when they are defined by crystal symmetry. An approximate (isotropic) treatment of cell e.s.d.'s is used for estimating e.s.d.'s involving l.s. planes.
Refinement. Refinement of *F*^2^ against ALL reflections. The weighted *R*-factor *wR* and goodness of fit *S* are based on *F*^2^, conventional *R*-factors *R* are based on *F*, with *F* set to zero for negative *F*^2^. The threshold expression of *F*^2^ > σ(*F*^2^) is used only for calculating *R*-factors(gt) *etc*. and is not relevant to the choice of reflections for refinement. *R*-factors based on *F*^2^ are statistically about twice as large as those based on *F*, and *R*- factors based on ALL data will be even larger.

### Fractional atomic coordinates and isotropic or equivalent isotropic displacement parameters (Å^2^)

**Table:**

	*x*	*y*	*z*	*U*_iso_*/*U*_eq_	
O1	0.1927 (2)	0.24083 (17)	0.16560 (15)	0.0730 (7)	
N1	0.2387 (2)	0.34793 (18)	0.31203 (16)	0.0491 (6)	
C1	0.2993 (4)	0.3817 (2)	0.4267 (2)	0.0633 (8)	
H1A	0.2614	0.3334	0.4799	0.076*	
H1B	0.4182	0.3838	0.4391	0.076*	
C2	0.2319 (4)	0.4866 (3)	0.4358 (3)	0.0842 (10)	
H2A	0.1506	0.4843	0.4863	0.101*	
H2B	0.3185	0.5349	0.4665	0.101*	
C3	0.1581 (4)	0.5259 (3)	0.3317 (3)	0.0819 (10)	
H3A	0.2195	0.5864	0.3098	0.098*	
H3B	0.0468	0.5490	0.3363	0.098*	
C4	0.1570 (3)	0.4378 (2)	0.2480 (2)	0.0620 (8)	
H4A	0.0457	0.4192	0.2170	0.074*	
H4B	0.2171	0.4585	0.1883	0.074*	
C5	0.2512 (3)	0.2526 (2)	0.2639 (2)	0.0457 (6)	
C6	0.3330 (3)	0.1583 (2)	0.33080 (19)	0.0429 (6)	
C7	0.2188 (3)	0.1215 (2)	0.4117 (2)	0.0551 (7)	
H7A	0.2114	0.1762	0.4660	0.083*	
H7B	0.2613	0.0574	0.4480	0.083*	
H7C	0.1118	0.1078	0.3720	0.083*	
C8	0.3515 (3)	0.0672 (2)	0.2504 (2)	0.0614 (8)	
H8A	0.4202	0.0898	0.1972	0.092*	
H8B	0.2455	0.0477	0.2127	0.092*	
H8C	0.4004	0.0069	0.2907	0.092*	
C9	0.5076 (3)	0.1884 (2)	0.38690 (19)	0.0414 (6)	
C10	0.5641 (3)	0.1624 (2)	0.4966 (2)	0.0530 (7)	
H10A	0.4940	0.1289	0.5394	0.064*	
C11	0.7227 (3)	0.1857 (2)	0.5426 (2)	0.0624 (8)	
H11A	0.7586	0.1675	0.6159	0.075*	
C12	0.8267 (3)	0.2348 (3)	0.4818 (3)	0.0654 (9)	
H12A	0.9334	0.2507	0.5132	0.078*	
C13	0.7727 (3)	0.2613 (3)	0.3722 (2)	0.0658 (9)	
H13A	0.8433	0.2947	0.3298	0.079*	
C14	0.6134 (3)	0.2378 (2)	0.3264 (2)	0.0561 (8)	
H14A	0.5778	0.2559	0.2530	0.067*	

### Atomic displacement parameters (Å^2^)

**Table:**

	*U*^11^	*U*^22^	*U*^33^	*U*^12^	*U*^13^	*U*^23^
O1	0.0851 (15)	0.0855 (15)	0.0406 (11)	0.0132 (12)	−0.0194 (10)	−0.0072 (11)
N1	0.0442 (13)	0.0590 (15)	0.0403 (12)	0.0075 (11)	−0.0073 (9)	0.0037 (11)
C1	0.079 (2)	0.0611 (19)	0.0433 (16)	0.0133 (17)	−0.0134 (14)	−0.0069 (14)
C2	0.104 (3)	0.076 (2)	0.070 (2)	0.027 (2)	0.0001 (19)	−0.0062 (18)
C3	0.095 (3)	0.062 (2)	0.082 (2)	0.0266 (19)	−0.0103 (19)	−0.0001 (19)
C4	0.0570 (18)	0.070 (2)	0.0553 (17)	0.0153 (15)	−0.0052 (14)	0.0161 (16)
C5	0.0368 (14)	0.0610 (18)	0.0364 (14)	0.0011 (13)	−0.0049 (11)	−0.0054 (14)
C6	0.0370 (14)	0.0497 (16)	0.0403 (14)	0.0045 (12)	−0.0009 (11)	−0.0053 (12)
C7	0.0466 (15)	0.0592 (18)	0.0589 (17)	−0.0028 (14)	0.0051 (13)	−0.0004 (14)
C8	0.0596 (18)	0.0657 (19)	0.0565 (17)	0.0064 (15)	−0.0005 (14)	−0.0222 (15)
C9	0.0386 (14)	0.0446 (15)	0.0386 (13)	0.0083 (12)	−0.0028 (11)	−0.0041 (12)
C10	0.0465 (15)	0.0663 (19)	0.0438 (15)	0.0067 (14)	−0.0016 (12)	0.0058 (14)
C11	0.0492 (17)	0.080 (2)	0.0519 (17)	0.0116 (16)	−0.0151 (14)	−0.0049 (16)
C12	0.0390 (16)	0.082 (2)	0.071 (2)	0.0110 (15)	−0.0092 (15)	−0.0231 (18)
C13	0.0397 (16)	0.087 (2)	0.071 (2)	−0.0079 (15)	0.0058 (14)	−0.0049 (18)
C14	0.0466 (16)	0.075 (2)	0.0453 (15)	−0.0055 (14)	0.0011 (12)	0.0074 (15)

### Geometric parameters (Å, º)

**Table:**

O1—C5	1.237 (3)	C6—C9	1.550 (3)
N1—C5	1.342 (3)	C7—H7A	0.9600
N1—C4	1.479 (3)	C7—H7B	0.9600
N1—C1	1.480 (3)	C7—H7C	0.9600
C1—C2	1.437 (4)	C8—H8A	0.9600
C1—H1A	0.9700	C8—H8B	0.9600
C1—H1B	0.9700	C8—H8C	0.9600
C2—C3	1.418 (4)	C9—C14	1.366 (3)
C2—H2A	0.9700	C9—C10	1.392 (3)
C2—H2B	0.9700	C10—C11	1.380 (4)
C3—C4	1.502 (4)	C10—H10A	0.9300
C3—H3A	0.9700	C11—C12	1.356 (4)
C3—H3B	0.9700	C11—H11A	0.9300
C4—H4A	0.9700	C12—C13	1.388 (4)
C4—H4B	0.9700	C12—H12A	0.9300
C5—C6	1.537 (3)	C13—C14	1.384 (3)
C6—C7	1.526 (3)	C13—H13A	0.9300
C6—C8	1.526 (3)	C14—H14A	0.9300
			
C5—N1—C4	120.3 (2)	C7—C6—C9	113.9 (2)
C5—N1—C1	129.2 (2)	C8—C6—C9	107.25 (19)
C4—N1—C1	110.5 (2)	C5—C6—C9	111.0 (2)
C2—C1—N1	104.6 (2)	C6—C7—H7A	109.5
C2—C1—H1A	110.8	C6—C7—H7B	109.5
N1—C1—H1A	110.8	H7A—C7—H7B	109.5
C2—C1—H1B	110.8	C6—C7—H7C	109.5
N1—C1—H1B	110.8	H7A—C7—H7C	109.5
H1A—C1—H1B	108.9	H7B—C7—H7C	109.5
C3—C2—C1	111.7 (3)	C6—C8—H8A	109.5
C3—C2—H2A	109.3	C6—C8—H8B	109.5
C1—C2—H2A	109.3	H8A—C8—H8B	109.5
C3—C2—H2B	109.3	C6—C8—H8C	109.5
C1—C2—H2B	109.3	H8A—C8—H8C	109.5
H2A—C2—H2B	107.9	H8B—C8—H8C	109.5
C2—C3—C4	108.4 (3)	C14—C9—C10	118.1 (2)
C2—C3—H3A	110.0	C14—C9—C6	119.6 (2)
C4—C3—H3A	110.0	C10—C9—C6	122.2 (2)
C2—C3—H3B	110.0	C11—C10—C9	120.9 (3)
C4—C3—H3B	110.0	C11—C10—H10A	119.6
H3A—C3—H3B	108.4	C9—C10—H10A	119.6
N1—C4—C3	104.0 (2)	C12—C11—C10	120.5 (3)
N1—C4—H4A	111.0	C12—C11—H11A	119.8
C3—C4—H4A	111.0	C10—C11—H11A	119.8
N1—C4—H4B	111.0	C11—C12—C13	119.5 (3)
C3—C4—H4B	111.0	C11—C12—H12A	120.2
H4A—C4—H4B	109.0	C13—C12—H12A	120.2
O1—C5—N1	119.1 (2)	C14—C13—C12	119.8 (3)
O1—C5—C6	120.4 (2)	C14—C13—H13A	120.1
N1—C5—C6	120.4 (2)	C12—C13—H13A	120.1
C7—C6—C8	108.3 (2)	C9—C14—C13	121.2 (3)
C7—C6—C5	108.2 (2)	C9—C14—H14A	119.4
C8—C6—C5	108.0 (2)	C13—C14—H14A	119.4
			
C5—N1—C1—C2	−172.4 (3)	N1—C5—C6—C9	−54.4 (3)
C4—N1—C1—C2	8.5 (3)	C7—C6—C9—C14	−170.0 (2)
N1—C1—C2—C3	−9.5 (4)	C8—C6—C9—C14	70.1 (3)
C1—C2—C3—C4	7.0 (4)	C5—C6—C9—C14	−47.7 (3)
C5—N1—C4—C3	176.3 (2)	C7—C6—C9—C10	13.0 (3)
C1—N1—C4—C3	−4.6 (3)	C8—C6—C9—C10	−106.8 (3)
C2—C3—C4—N1	−1.3 (4)	C5—C6—C9—C10	135.4 (2)
C4—N1—C5—O1	−0.5 (4)	C14—C9—C10—C11	−0.2 (4)
C1—N1—C5—O1	−179.4 (3)	C6—C9—C10—C11	176.8 (2)
C4—N1—C5—C6	−178.8 (2)	C9—C10—C11—C12	0.3 (4)
C1—N1—C5—C6	2.2 (4)	C10—C11—C12—C13	−0.3 (5)
O1—C5—C6—C7	−107.0 (3)	C11—C12—C13—C14	0.3 (5)
N1—C5—C6—C7	71.3 (3)	C10—C9—C14—C13	0.2 (4)
O1—C5—C6—C8	10.0 (3)	C6—C9—C14—C13	−176.9 (3)
N1—C5—C6—C8	−171.7 (2)	C12—C13—C14—C9	−0.2 (4)
O1—C5—C6—C9	127.3 (2)		

### Hydrogen-bond geometry (Å, º)

**Table:**

*D*—H···*A*	*D*—H	H···*A*	*D*···*A*	*D*—H···*A*
C1—H1*A*···O1^i^	0.97	2.58	3.510 (4)	160

Symmetry code: (i) *x*, −*y*+1/2, *z*+1/2.
